# Implementation of *Choosing Wisely*^®^ Recommendations for Lymph Node Surgery in Male Breast Cancer

**DOI:** 10.1245/s10434-024-15811-x

**Published:** 2024-07-20

**Authors:** Catherine G. Pratt, Jenna N. Whitrock, Michela M. Carter, Szu-Aun Long, Jaime D. Lewis, Alicia A. Heelan

**Affiliations:** 1 Cincinnati Research in Outcomes and Safety in Surgery (CROSS) Research Group, Department of Surgery, University of Cincinnati College of Medicine, Cincinnati, OH USA; 2 Division of Surgical Oncology, Department of Surgery, University of Cincinnati College of Medicine, Cincinnati, OH USA

**Keywords:** Male breast cancer, Breast cancer, *Choosing Wisely*^®^, Lymph node, Sentinel lymph node biopsy, Axillary lymph node dissection

## Abstract

**Background:**

The *Choosing Wisely*^®^ (*CW*) campaign recommended de-implementation of surgical management of axillary nodes in specified patients. This study aimed to assess trends in the application of *CW* guidelines for lymph node (LN) surgery in males with breast cancer.

**Methods:**

The National Cancer Database was queried for males diagnosed with breast cancer from 2017 to 2020. Patients were categorized into two cohorts based on *CW* criteria. Cohort 1 included all T1-2, clinically node-negative patients who underwent breast-conserving therapy and with ≤ 2 positive nodes, and Cohort 2 included all T1-2, node-negative, hormone receptor-positive, human epidermal growth factor receptor 2 (HER2)-negative patients aged ≥ 70 years. In Cohort 1, patients who underwent sentinel LN biopsy (SLNB) alone were compared with axillary LN dissection (ALND) or no LN surgery, while in Cohort 2, patients who underwent LN surgery were compared with those with no LN surgery.

**Results:**

Of 617 patients who met the criteria for Cohort 1, 73.1% underwent SLNB alone compared with ALND (11.8%) or no LN surgery (15.1%). Those who received SLNB alone were younger (65 vs. 68 vs. 73 years; *p* < 0.001). The annual proportion of males who underwent SLNB alone remained stable from 2017 to 2020. Overall, 1565 patients met the criteria for Cohort 2, and 84.9% received LN surgery. LN surgery was omitted in older patients (81 vs. 77; *p* < 0.001). The proportion of elderly males with early-stage breast cancer who underwent LN surgery increased from 2017 to 2020.

**Conclusion:**

This study demonstrates that *CW* recommendations are not being routinely applied to males. These findings reinforce the need for additional studies and subsequent recommendations for optimal application of axillary surgery de-implementation for males diagnosed with breast cancer.

In 2016, the national *Choosing Wisely*^®^* (CW)* campaign was founded by the American Board of Internal Medicine Foundation to address overtreatment and low-value care in medicine.^1^ Low-value care consists of medical services that hold minimal to no clinical benefit but deliver cost and associated risks to patients.^2^ As part of this campaign, 17 surgical societies participated and generated over 110 recommendations to minimize overtreatment in surgery.^3^ Most campaign recommendations targeted perioperative care; however, the recommendations regarding breast cancer treatment were an exception.

Together, the American College of Surgeons, the Society for Surgical Oncology, and the American Society for Breast Surgeons utilized randomized clinical trials and meta-analyses to identify and recommend the de-implementation of low-value procedures for breast cancer. The four identified procedures included (1) axillary lymph node dissection (ALND) for limited nodal disease in patients receiving breast-conserving therapy (based on the American College of Surgeons Oncology Group [ACOSOG] Z0011 trial);^4^ (2) re-excision for close but negative margins for invasive cancer;^5,6^ (3) contralateral prophylactic mastectomy in average-risk patients with unilateral cancer;^7^ and (4) sentinel lymph node biopsy (SLNB) in women aged ≥ 70 years with early-stage, hormone receptor-positive (HR+), human epidermal growth factor receptor 2-negative (HER2−) cancer.^8,9^ It was determined that these procedures did not increase overall survival (OS) among breast cancer patients, but conferred both increased medical costs and the risk of associated postoperative morbidity.^9^ These campaign recommendations have shaped contemporary guidelines for the management of breast cancer. The application of these guidelines for females has proven feasible and has resulted in overall de-implementation;^10–12^ however, guideline-directed management and oncologic outcomes differ for male breast cancer (MBC).^13,14^

Despite the overall prevalence of breast cancer worldwide, breast cancer in males is rare, representing < 1% of cases.^15^ As a result, MBC is historically understudied and poorly represented in the literature. Consequently, guidelines for treatment have been extrapolated from female cohorts, and the National Comprehensive Cancer Network (NCCN) recommends that a similar approach to postmenopausal female management be applied to MBC patients, including the approach to axillary surgery management.^15,16^

Considering the paucity of studies conducted on MBC and limited evidence to direct management, understanding treatment trends and outcomes in these patients is of interest. Literature reviews of male patients with breast cancer reveal that surgical management and outcomes diverge from their female counterparts, particularly with a higher utilization of invasive approaches.^15,17–19^ While limited studies have been conducted to evaluate the de-implementation of *CW* procedures for females,^10–12^ no analogous studies have evaluated their application in male patients. The purpose of this study was to employ a nationwide, multicenter database to assess trends in the application of recommendations for de-implementation of lymph node (LN) surgery in males with breast cancer. We hypothesized that the *CW* guidelines are infrequently considered in LN surgery in MBC and that de-implementation rates are low.

## Methods

### Data Source and Study Population

Data from the National Cancer Database (NCDB), a joint initiative between the American College of Surgeons’ and the American Cancer Society, were utilized for this study. As it includes more than 1500 Commission on Cancer (CoC)-accredited facilities, this nationwide oncology database captures 70% of newly diagnosed cancer cases within the United States. The NCDB reports data on facility characteristics, patient demographics, tumor characteristics, treatments, and outcomes.^20,21^ Data from the NCDB are de-identified, thus this work was deemed exempt from Institutional Review Board review (IRB#2024-0367).

The NCDB was queried for all males diagnosed with breast cancer from 2017 to 2020. Patients were included if they had invasive ductal carcinoma (IDC) or lobular carcinoma. Exclusion criteria included female patients; patients with unknown regional LN management; patients who did not receive surgical intervention; patients for whom radiation was not recommended or was contraindicated; patients who refused radiation; patients who received neoadjuvant therapy or intraoperative radiation; patients for whom the radiation treatment sequence was unknown; patients diagnosed with ductal carcinoma in situ alone; patients with unknown T stage; patients with unknown N stage; or patients with clinical M stage > 0.

**Fig. 1:**
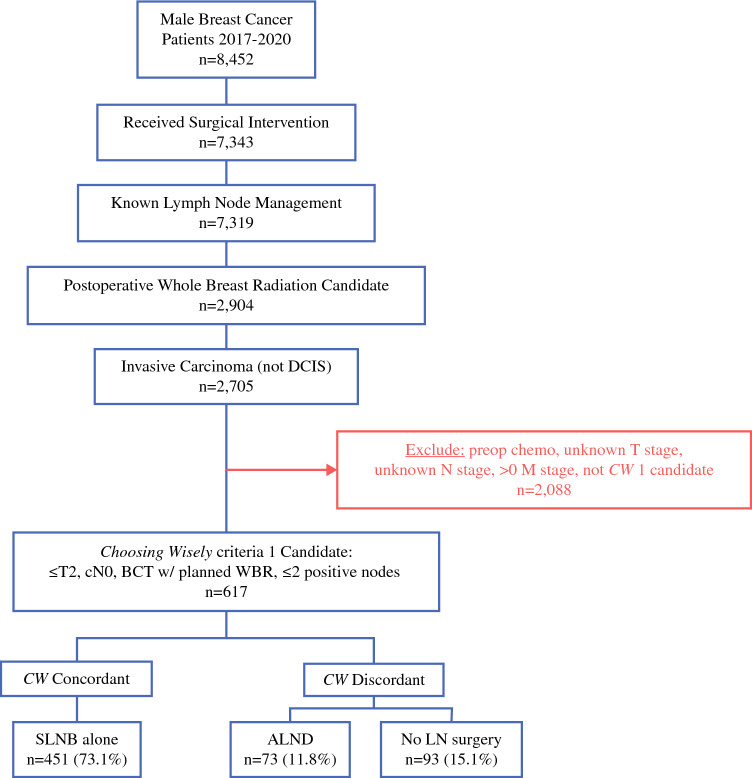
Cohort 1: Exclusions and grouping of NCDB male breast cancer patients from 2017 to 2020. *BCT* breast-conserving therapy, *WBR* whole-breast radiation, *SLNB* sentinel lymph node biopsy, *ALND* axillary lymph node dissection, *LN* lymph node, *DCIS* ductal carcinoma in situ, *CW* *Choosing Wisely*^®^, *preop chemo* preoperative chemotherapy, *NCDB* National Cancer Database

**Fig. 2:**
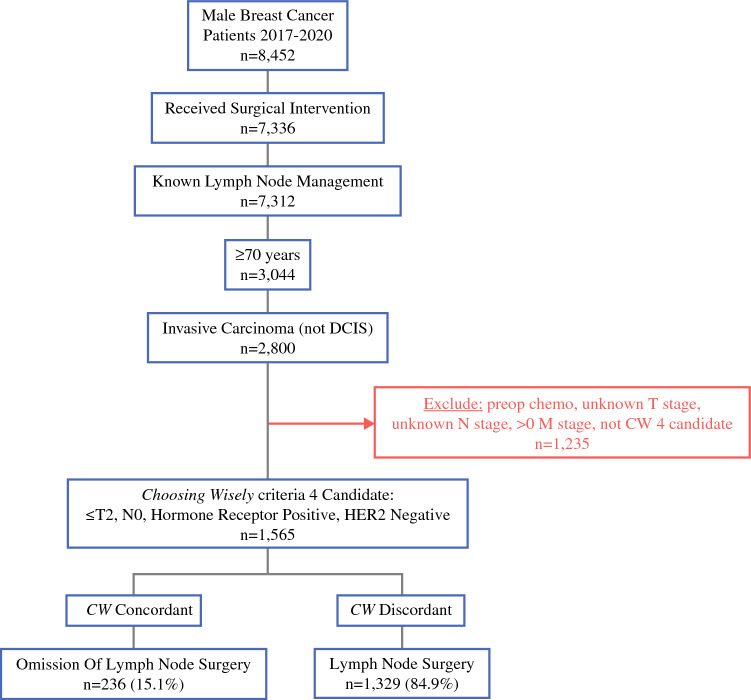
Cohort 2: Exclusions and grouping of NCDB male breast cancer patients ≥ 70 years of age from 2017 to 2020. *DCIS* ductal carcinoma in situ, *CW* *Choosing Wisely*^®^, *preop chemo* preoperative chemotherapy, *HER2* human epidermal growth factor receptor, *NCDB* National Cancer Database

Patients were categorized into two cohorts based on *CW* criteria (1) and (4), as defined above. Cohort 1 included all patients with T1-2, clinically node-negative breast cancer who underwent breast-conserving therapy (BCT) with planned whole-breast radiation and ≤ 2 positive nodes. BCT was defined as lumpectomy, partial mastectomy, or segmental mastectomy followed by radiation. Cohort 1 was further divided into patients who underwent SLNB alone (*CW*-concordant) versus patients who underwent ALND or no LN surgery (*CW*-discordant) to serve as comparison groups (Fig. 1). Cohort 2 included all patients ≥ 70 years of age with T1-2, node-negative, HR+, HER2− breast cancer. HR+ was defined as estrogen receptor- and/or progesterone receptor-positive on histology. Cohort 2 was further divided into patients with omission of LN surgery (*CW*-concordant) versus patients who underwent LN surgery (*CW*-discordant) [Fig. 2]. Given the inclusion criteria, Cohorts 1 and 2 are not mutually exclusive.

### Data Variables

Baseline characteristics of all patients were examined, including age, race, treatment facility type, insurance status, geographical location size, and Charlson–Deyo score. Tumor histology was analyzed utilizing the NCDB-supplied classifications of ductal, lobular, mixed, or other (unspecified invasive carcinoma). These factors and temporal trends in management approach were evaluated for each cohort. In Cohorts 1 and 2, *CW*-concordant patients were compared with *CW*-discordant patients, as defined above.

### Statistical Analysis

Continuous variables were compared using the Kruskal–Wallis or Wilcoxon rank-sum tests, as appropriate. Pearson Chi-square tests were utilized for analysis of categorical variables. Continuous variables were described using mean (standard deviation) or median (interquartile range [IQR]), and categorical variables were summarized using frequencies and percentages, as appropriate. If incomplete data were noted for a case entry, that case was excluded from the specific analysis for which data were unavailable. Mean OS for patients were compared using Kaplan–Meier analysis. OS was defined as time from the date of diagnosis until the date of reported death or last documented patient contact. All statistical analyses were performed using JMP Pro v16 (SAS Institute, Cary, NC, USA).

## Results

### Cohort 1: Population Characteristics

Between 2017 and 2020, 617 males were identified within the NCDB as having been diagnosed with breast cancer and met the study inclusion criteria for Cohort 1. Of these 617 males, 451 (73.1%) underwent SLNB alone, 73 (11.8%) underwent ALND, and 93 (15.1%) underwent no LN surgery. Table 1 summarizes the overall baseline characteristics and between-group characteristic comparison for Cohort 1. Median age [IQR] was 66 years [59–73]. 83.3% of patients identified as White, 12.2% identified as Black, and 4.4% identified as other or unknown race. Most patients received care at comprehensive community cancer programs (46.5%), fewer received treatment at academic/research programs (22.6%) or integrated network cancer programs (21.6%), and very few received care at community cancer programs (0.4%). When evaluating insurance status, most patients had Medicare (53.3%) or private insurance (40.7%), while fewer had Medicaid/other government insurance (4.6%) or were uninsured (1.5%). Most patients were in a metropolitan setting (86.3%) compared with a minority in either urban (11.7%) or rural (2.0%) settings. When evaluating Carlson–Deyo score as a surrogate for comorbidities, patients most frequently scored a 0 (76.7%), while fewer scored a 1 (14.3%), and very few scored a 2 (4.9%) or ≥ 3 (4.2%). Upon evaluation of histology, most patients had ductal carcinoma (82.3%) and fewer patients had lobular (6.2%), mixed (5.5%), or other (6.0%).

**Table 1:** Cohort 1: patient characteristics and group comparisons between SLNB alone, ALND, and no LN surgery

Category	Overall [*n* = 617]	SLNB alone [*n* = 451 (73.1%)]	ALND [*n* = 73 (11.8%)]	No LN surgery [*n* = 93 (15.1%)]	*p*-value
Age, years (median [IQR])	66 [59–73]	65 [58–72]	68 [58.5–77.6]	73 [64.5–85.6]	< 0.001*
Race					0.11
White	511 (83.3)	377 (84.2)	64 (88.9)	70 (75.3)	
Black	75 (12.2)	50 (11.2)	7 (9.7)	18 (19.4)	
Other	27 (4.4)	21 (4.7)	1 (1.4)	5 (5.4)	
Facility type					0.85
CCP	57 (0.4)	43 (9.7)	8 (11.1)	6 (6.5)	
Comprehensive CCP	282 (46.5)	206 (46.5)	35 (48.6)	41 (44.6)	
Academic/research program	137 (22.6)	102 (23.0)	13 (18.1)	22 (23.9)	
Integrated network cancer program	131 (21.6)	92 (20.8)	16 (22.2)	23 (25.0)	
Insurance status					0.004*
Not insured	9 (1.5)	7 (1.6)	2 (2.8)	0 (0)	
Private insurance	248 (40.7)	195 (43.8)	30 (41.7)	23 (24.7)	
Medicaid/other government	28 (4.6)	21 (4.7)	5 (6.9)	2 (2.2)	
Medicare	325 (53.3)	222 (49.9)	35 (48.6)	68 (73.1)	
Location size					0.94
Metro	525 (86.3)	385 (86.9)	60 (83.3)	80 (86.0)	
Urban	71 (11.7)	50 (11.3)	10 (13.9)	11 (11.8)	
Rural	12 (2.0)	8 (1.8)	2 (2.8)	2 (2.2)	
Charlson–Deyo score					0.12
0	473 (76.7)	350 (77.6)	57 (78.1)	66 (71.0)	
1	88 (14.3)	65 (14.4)	12 (16.4)	11 (11.8)	
2	30 (4.9)	19 (4.2)	3 (4.1)	8 (8.6)	
≥ 3	26 (4.2)	17 (3.8)	1 (1.4)	8 (8.6)	
Histology					< 0.001*
Ductal	508 (82.3)	380 (84.3)	62 (84.9)	66 (71.0)	
Lobular	38 (6.2)	32 (7.1)	3 (4.1)	3 (3.2)	
Mixed	34 (5.5)	20 (4.4)	3 (4.1)	11 (11.8)	
Other	37 (6.0)	19 (4.2)	5 (6.8)	13 (14.0)	

Data are expressed as *n* (%) unless otherwise specified

*LN* lymph node, *SLNB* sentinel LN biopsy, *ALND* axillary LN dissection, *IQR* interquartile range, *CCP* community cancer program

*Indicates statistical significance

### Cohort 1: Group Characteristic Comparison

Among patient demographic variables evaluated, patient age and insurance status were significantly associated with approach to LN management in male patients with clinically node-negative breast cancer. Patients who received SLNB alone and ALND were younger (65 [58–72] and 68 [58.5–77.6] years) compared with no LN surgery (73 [64.5–85.6] years; *p* < 0.001). MBC patients who underwent SLNB alone or ALND were more likely to have private insurance (43.8% vs. 41.7%; *p* = 0.004) than patients who received no LN surgery (24.7%). Those who received ALND were more likely to be uninsured (2.8%) or have non-Medicare, Government-sponsored insurance (6.9%) than patients who received SLNB alone (1.6% and 4.7%, respectively) or no LN surgery (0% and 2.2%, respectively) [*p* = 0.004 for all]. There was no significant difference between groups regarding race, type of treatment facility, geographical location size, or Charlson–Deyo score. Patients in whom LN surgery was omitted were less likely to have IDC on histology than those who received SLNB alone or ALND (71.0% vs. 84.3% vs. 84.9%) and were more likely to have mixed (11.8% vs. 4.4% vs. 4.1%) or other (14.0% vs. 4.2% vs. 6.8%) breast cancer histology (*p* = 0.0004).

### Cohort 1: Temporal Trends in Lymph Node Management

**Fig. 3:**
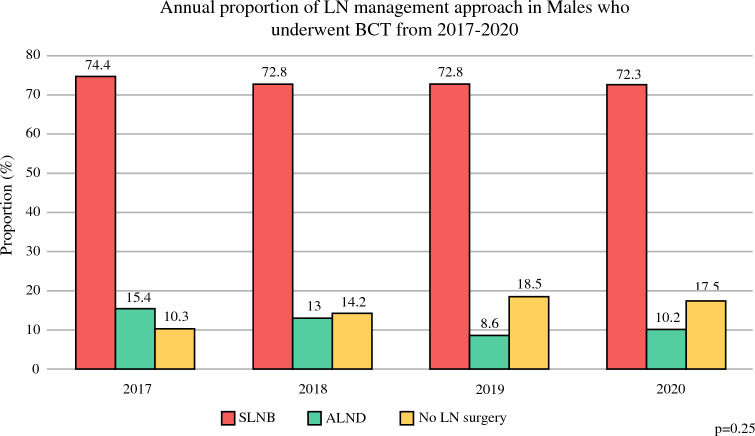
Cohort 1: LN management by year. *LN* lymph node, *BCT* breast-conserving therapy, *SLNB* sentinel lymph node biopsy, *ALND* axillary lymph node dissection

Upon evaluation of temporal trends, there was no significant change in LN management over time (*p* > 0.05) (Fig. 3). *CW-*concordant management of SLNB alone was received by 74.4% of males in 2017 compared with 72.8% in 2018, 72.8% in 2019, and 72.3% in 2020. Rates of ALND and omission of LN surgery (*CW*-discordant) also remained stable, however their relative annual proportions reversed over the study period. ALND decreased from 15.4% in 2017 to 10.2% in 2020. Conversely, omission of LN surgery increased from 10.3% in 2017 to 17.5% in 2020.

### Cohort 2 Population Characteristics

Between 2017 and 2020, 1565 males were identified within the NCDB as having been diagnosed with breast cancer and met the study inclusion criteria for Cohort 2. Of these 1565 patients, 236 (15.1%) had omission of LN surgery and 1329 (84.9%) underwent LN surgery. Most (74.6%) LN surgery recipients received SLNB alone. Table 2 summarizes the overall baseline characteristics and between-group characteristic comparison for Cohort 2. Median age [IQR] was 77 [73–82] years. 88.3% of patients identified as White, 8.7% identified as Black, and 3.0% identified as other or unknown race. Most patients received care at comprehensive community cancer programs (43.5%), fewer received treatment at academic/research programs (27.0%) or integrated network cancer programs (20.0%), while very few received care at community cancer programs (9.4%). When evaluating insurance status, most patients had Medicare (86.7%), while fewer had private insurance (9.9%) or Medicaid/other government insurance (2.8%), and very few were uninsured (0.7%). Most patients were in a metropolitan setting (86.8%) compared with a minority in either urban (11.7%) or rural (1.5%) settings. When evaluating Carlson–Deyo score, most patients scored a 0 (68.3%), while fewer scored a 1 (16.9%), and very few scored a 2 (8.0%) or ≥ 3 (6.8%). Patients in whom LN surgery was omitted were less likely to have IDC on histology than those who received SLNB alone or ALND (81.4% vs. 87.9%) and were more likely to have mixed (5.5% vs. 2.4%) or other (10.6% vs. 7.2%) breast cancer histology (*p* = 0.014).

**Table 2:** Cohort 2: Patient characteristics and group comparisons between omission of LN surgery and LN surgery

Category	Overall [*n* = 1565]	Omission of LN surgery [*n* = 236 (15.1%)]	LN surgery [*n* = 1329 (84.9%)]	*p*-Value
Age, years (median [IQR])	77 [73–82]	81 [76–87]	77 [73–81]	< 0.001*
Race				0.06
White	1376 (88.3)	1171 (88.6)	205 (86.9)	
Black	135 (8.7)	117 (8.9)	18 (7.6)	
Other	47 (3.0)	34 (2.6)	13 (5.5)	
Facility type				0.85
CCP	148 (9.4)	125 (9.4)	23 (9.8)	
Comprehensive CCP	681 (43.5)	583 (43.9)	98 (41.5)	
Academic/research program	423 (27.0)	354 (26.6)	69 (29.2)	
Integrated network cancer program	313 (20.0)	267 (20.1)	46 (19.5)	
Insurance status				0.37
Not insured	10 (0.7)	10 (0.8)	0 (0)	
Private Insurance	153 (9.9)	131 (9.9)	22(9.5)	
Medicaid/other government	44 (2.8)	40 (3.0)	4 (1.7)	
Medicare	1343 (86.7)	1137 (86.3)	206 (88.8)	
Location size				0.96
Metro	1337 (86.8)	1135 (86.8)	202 (86.8)	
Urban	179 (11.6)	153 (11.7)	26 (11.2)	
Rural	24 (1.6)	20 (1.5)	4 (1.7)	
Charlson–Deyo score				0.35
0	1069 (68.3)	910 (68.5)	159 (68.3)	
1	264 (16.9)	230 (17.3)	35 (14.4)	
2	125 (8.0)	101 (7.6)	24 (10.2)	
≥ 3	107 (6.8)	88 (6.6)	19 (8.1)	
Histology				0.014*
Ductal	1360 (86.9)	1168 (87.9)	192 (81.4)	
Lobular	39 (2.5)	33 (2.5)	6 (2.5)	
Mixed	45 (2.9)	32 (2.4)	13 (5.5)	
Other	121 (7.7)	96 (7.2)	25 (10.6)	

Data are expressed as *n* (%) unless otherwise specified

*LN* lymph node, *IQR* interquartile range, *CCP* community cancer program

*Indicates statistical significance

### Cohort 2: Group Characteristic Comparison

Among patient demographic variables evaluated, patient age was significantly associated with approach to LN management in elderly male patients with early-stage, node-negative breast cancer. Males ≥ 70 years of age for whom LN surgery was omitted were older (81 [76–87] years; *p* < 0.001) compared with males who underwent LN surgery (77 [73–81] years). There was no significant difference between groups regarding race, type of treatment facility, insurance status, geographical location size, or Charlson–Deyo score.

### Cohort 2: Temporal Trends in Lymph Node Management

**Fig. 4:**
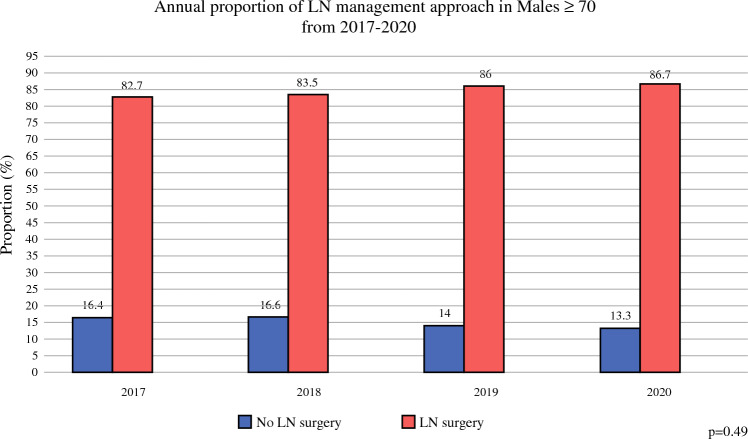
Cohort 2: LN management by year. *LN* lymph node

Upon evaluation of temporal trends, the proportion of elderly males with early-stage breast cancer who underwent LN surgery increased over time (Fig. 4). *CW-*concordant management of omission of LN surgery decreased from 17.3% of males in 2017 to 13.8% in 2020. In contrast, rates of any LN surgery (*CW*-discordant) increased from 82.7% in 2017 to 86.7% in 2020. Overall, this trend did not represent a statistically significant change between the groups during the study period (*p* > 0.05).

### Survival Analysis

**Fig. 5:**
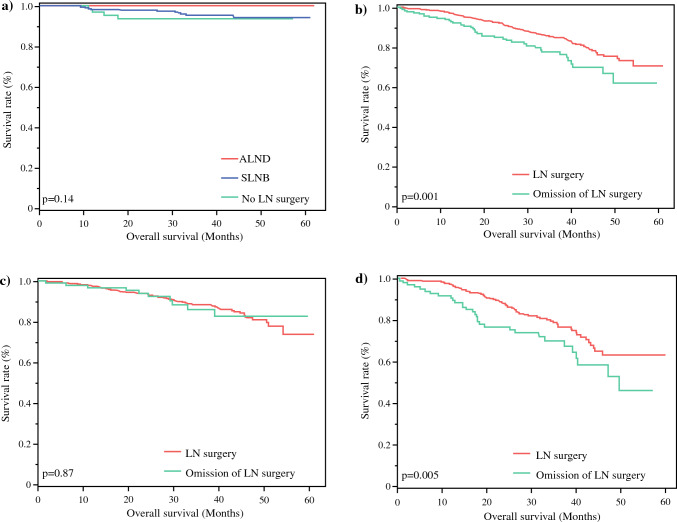
(**a**) Cohort 1: Kaplan–Meier curve comparing patient overall survival between SLNB alone, ALND, and no LN surgery. (**b**) Cohort 2: Kaplan–Meier curve comparing patient overall survival between omission of LN surgery and LN surgery for patients ≥ 70 years of age. (**c**) Stratified Cohort 2: Kaplan–Meier curve comparing patient overall survival between omission of LN surgery and LN surgery for patients aged 70–80 years. (**d**) Stratified Cohort 2: Kaplan–Meier curve comparing patient overall survival between omission of LN surgery and LN surgery for patients aged 80+ years. *ALND* axillary lymph node dissection, *SLNB* sentinel lymph node biopsy, *LN* lymph node

Kaplan–Meier analysis of Cohort 1 comparing SLNB alone, ALND, and no LN surgery found no significant difference in OS (*p* = 0.14) (Fig. 5a). Only 75% of Cohort 1 patients had recent follow-up documented (75% SLNB, 60% ALND, 80% no LN surgery). No mean survival was able to be calculated secondary to the paucity of recorded patient deaths.

Kaplan–Meier analysis of Cohort 2 comparing omission of LN surgery with LN surgery found a significant difference in OS (*p* = 0.001) (Fig. 5b). Only 66% of Cohort 2 patients had recent follow-up documented (63% omission of LN surgery, 66% LN surgery). Following stratification of Cohort 2 into age groups of 70–80 years and ≥ 81 years, no significant difference in OS for the 70–80 years age group was found on subsequent survival analyses (*p* = 0.87) (Fig. 5c); however, a significant difference in OS for patients ≥ 81 years of age was detected (*p* = 0.005) (Fig. 5d). No mean survival was able to be calculated due to limited recorded patient deaths.

## Discussion

According to the American Cancer Society, an estimated 2800 males were diagnosed with breast cancer in 2023.^22^ The published literature regarding management approaches to MBC is limited to observational or retrospective studies and no current reviews evaluate treatment strategies from a *CW* guideline approach.^23^ Initial publication of the *CW* recommendations in September 2016 changed practice patterns for the management of breast cancer in females. Despite this, evidence has demonstrated a poor application of these guidelines to male patients despite the increased cost and risk of unnecessary invasive procedures.^23–26^ Therefore, the primary objective of this study was to evaluate trends of LN management for MBC using a large nationwide database (NCDB) through the lens of the *CW* guidelines. These data found that LN management strategies for clinically node-negative MBC have not changed in the years following publication of the *CW* recommendations. Furthermore, LN management has trended away from *CW* recommendations among elderly males diagnosed with early-stage, node-negative breast cancer.

### *Choosing Wisely*^®^ (*CW*) Criteria 1/Cohort 1: T1-2, Clinically Node-Negative Male Breast Cancer Patients Undergoing Breast-Conserving Therapy with ≤ 2 Positive Lymph Nodes

Among male patients with breast cancer, partial mastectomy has been associated with a reduction in mortality risk.^23,24^ Even so, no change in management strategy for males has been exhibited over time. In a retrospective cohort study, Cardoso et al.^24^ demonstrated that while the majority of MBCs diagnosed between 1990 and 2010 were early-stage disease, only 4% received BCT. A systematic review including eight studies from 1998 to 2016 revealed that the de-implementation of surgical intervention has not occurred for MBC.^26^ More recently, Singh et al.^26^ again demonstrated significantly lower rates of partial mastectomy in males compared with females in an NCDB analysis of clinical T1-2 breast cancers diagnosed between 2004 and 2016. In a related study, Carter et al.^27^ found no significant change in management approach for male patients with ductal carcinoma in situ from 2006 to 2017.

Similarly, we found low rates of BCT (617 patients) overall in our NCDB data query in the 4 years immediately following the publication of *CW* recommendations. In this population of males who underwent BCT, the majority (73.1%) received SLNB, which is concordant with *CW* recommendations. However, there was no significant decrease in the rate of ALND over the study period. While SLNB has been demonstrated as safe and efficacious for MBC in appropriate patients,^28^ the lack of change in clinical practice regarding ALND is concerning considering the associated morbidity. As this study found no significant difference in OS between axillary management approaches in Cohort 1, this could be interpreted as further, although limited, endorsement for forgoing ALND in this group. Since publication of ACOSOG Z011, there has been wide acceptance of omission of ALND in early-stage female breast cancer with low-volume nodal disease.^4,10–12^ In fact, de-implementation of ALND has even been extended to patients with nodal disease that is cleared with neoadjuvant chemotherapy. While it is encouraging that most male patients within the small population receiving BCT appear to undergo Z011-directed axillary management, the persistent use of ALND or complete omission of LN surgery may indicate a hesitancy to apply this practice change to males.

Additionally, it is notable that we found a difference in the median age between male patients who received SLNB or ALND, and those who had complete omission of LN surgery. When comparing the median age of SLNB alone with ALND, no significant difference was found. However, when considering the median age of MBC patients for those who had no LN surgery, a significantly older age was noted. This trend of electing for less-aggressive measures in an older patient has been described previously in breast cancer management. Carter et al. describe a younger median age for male patients who underwent mastectomy with radiation after diagnosis with ductal carcinoma in situ compared with partial mastectomy alone, partial mastectomy with radiation, or mastectomy alone.^27^ Furthermore, in an evaluation of axillary management in early-stage, clinically node-negative women found to have one to three positive nodes on SLNB during upfront surgery, Tadros et al.^29^ found an older median age was associated with less aggressive management in both choice of primary tumor management (BCT vs. mastectomy) and axillary treatment following surgery. While the exact reasons behind management choice cannot be determined from the NCDB, we suspect that more extensive measures are considered for younger patients due to a combination of life expectancy, physical fitness, the ability to tolerate therapy, and any indication of aggressive tumor biology.

### *CW* Criteria 4/Cohort 2: ≥ 70 Years of Age with Node-Negative, Hormone Receptor-Positive, Human Epidermal Growth Factor Receptor 2-Negative Male Breast Cancer

In contrast to the other procedures recommended for de-implementation by the *CW* campaign, providers have proven resistant to championing the final guideline for females with breast cancer aged ≥ 70 years.^30^ Likewise, these data demonstrate the majority (84.9%) of MBC patients ≥ 70 years of age with early-stage clinically node-negative, HR+, HER2− cancer underwent some form of LN intervention. Furthermore, this high rate of intervention has had a mild increase, although it fails to reach statistical significance, following *CW* guideline publication. The *CW* campaign cites studies that support that the omission of SLNB in this population does not affect patient treatment decisions or outcomes and only increases morbidity and risk.^1,8, 9^ Few data exist on why clinicians continue to pursue invasive LN management in the female contingent of this patient population.^31^ So too, a paucity of previous data on MBC among patients ≥ 70 years of age exist to reference for similar findings. Beyond offering or omitting axillary surgery, the extent of other locoregional or adjuvant therapies in the study population is unknown due to the limitations of the NCDB, which are discussed below. The current lack of research and society-endorsed guidelines for MBC creates an environment where management choice for all treatment modalities is widely variable and leaves providers without definitive direction for these patients. Additional work is needed to understand why clinicians forgo guideline-directed treatment among females and to direct providers when treating males in this age category.

Attention should be given to the survival analyses for Cohort 2. While recommending caution in interpretation considering the small number of patient deaths recorded, some differences in OS were identified in this study. Regarding the long OS found among patients ≥ 70 years of age who received LN surgery, this appears to be influenced primarily by the patients aged ≥ 81 years. As demonstrated in the stratified survival analysis, a significant OS difference persisted only between this oldest group of male patients. As this analysis considers overall survival, it is impossible to interpret whether the survival difference detected is secondary to cancer-related causes. Generally, breast cancer-specific survival in these populations would be expected to be high and most recurrence found at >10 years after initial diagnosis, thus these survival analyses are unlikely to reflect cancer-related mortality. Especially in this oldest patient population most at risk for comorbid conditions and with lower expected life expectancy, limited application to axillary management strategies can be made.

### Female Breast Cancer Compared with Male Breast Cancer

Although management of MBC has predominantly been extrapolated from studies on female patients, notable differences have been identified. On average, male patients present at an older age, with more advanced stage, and higher rates of HR positivity.^25,32^ Even so, MBC presents with a similar predominance of IDC and appears biologically similar to postmenopausal HR+ disease in females.^16,32^ Comparably, this study found most patients presented with IDC histology cohorts.

Promisingly, mortality has improved over time in both males and females, which is primarily attributed to improvements in adjuvant therapy; however, MBC mortality has experienced a slower improvement.^14^ Li et al.^33^ found that early-stage MBC diagnosed from 2010 to 2012 had significantly worse overall survival than stage-matched female patients, when adjusting for age, ethnicity, and tumor grade. Based on this, Li et al. posited that MBC may necessitate more aggressive treatment than female breast cancer. Conversely, when evaluating treatment approach in early-stage MBC diagnosed from 2004 to 2016, Singh et al.^26^ reported that males consistently receive more extensive surgery than females, including higher rates of mastectomy and ALND. In the same study, partial mastectomy was found to confer a 42% reduction in mortality risk for males.^26^ Similarly, the findings in this study demonstrate high rates of more extensive surgical intervention than recommended in the post-*CW* era. Considering the lag in MBC mortality improvement, despite persistent use of extensive intervention, de-implementation of low-value care in MBC should be emphasized.

### Resistance to the Adoption of *CW* Guidelines in Male Breast Cancer

Several reasons may be connected to delayed de-implementation for MBC patients and are worth noting. First, the lingering belief in differing biology and/or need for aggressive treatment. As discussed previously, this has not been proven in the current literature or in overall survival for MBC. Second, questions remain regarding the effectiveness of adjuvant treatment (specifically endocrine therapy). Indeed, prior reports indicated lower utilization of adjuvant endocrine therapy in MBC.^34–36^ However, despite the absence of male-specific clinical trials, underutilization of adjuvant treatment has been associated with the slower improvement in MBC survival.^11,35, 36^ Third, clinicians’ concerns regarding the scarcity of data in MBC management and a corresponding lack of support in medical-legal standings for non-guideline-based care. While the medical-legal argument is debatable, a patient-centered personalized care approach is best from a patient outcomes and legal standpoint. Fourth, a lack of awareness of *CW* recommendations and applicability to MBC. Since publication, the *CW* recommendations are increasingly being recognized in medical and surgical communities.^36^ While it is possible that center-specific treatment approaches differ, this study found similar rates of *CW*-concordant and -discordant LN management across all treatment facility types. Furthermore, the *CW* identification of low-value procedures for breast cancer were made in part by the American College of Surgeons (ACS). Therefore, with the CoC being an ACS initiative, the NCDB included only CoC-accredited hospitals where the treating clinicians would have an expected understanding of ACS-endorsed guidelines for breast cancer care. Even so, recognition alone has not proven sufficient to result in a change in clinical practice.^37^ Studies that have demonstrated the highest rates of *CW*-concordant de-implementation demonstrate organizational-level change involving the multiple procedures.^10,11, 37^ Considering this, the lack of *CW* concordance in LN management among CoC-accredited hospitals is particularly striking.

### Limitations

Limitations of this study exist that warrant discussion. First, NCDB is a retrospective multi-institutional database and therefore limitations inherent to utilization apply to this study. This includes the inability to reference the source data, which limited the investigation to only what data were provided. The absence of such data makes some important analyses impossible, such as comorbidities, family history, and genetic testing. These factors are integral when deciding the optimal management approach for patients with breast cancer. Furthermore, outcome analysis afforded by the NCDB is limited to overall survival for patients for whom a recent follow-up has been documented. As such, cancer-specific and long-term outcomes based on an axillary management strategy are not able to be assessed. Any interpretation or application of OS in these cohorts should be interpreted with caution, as discussed above. Additionally, the use of a large database predisposes to potential miscoding. However, the benefits of a large, multi-institutional database and the associated patient volume were felt to outweigh such risks. This is particularly true for investigating MBC patients, as the disease’s rarity results in a lack of high-powered studies. Furthermore, NCDB is limited to data from CoC-accredited hospitals. Such institutions may incompletely account for national trends and may not reflect practice patterns at non-CoC-accredited facilities.

This is the first study to utilize a nationwide, multi-institutional database to evaluate application of *CW* guidelines to MBC. While the results are informative, the reasons for lack of implementation in this population are not obvious and are likely multifactorial. Further studies are required to elucidate the reasons for these trends in MBC care and to better understand the optimal management strategy for this patient population.

## Conclusions

The data from this multi-institutional nationwide database study demonstrate males with breast cancer are undergoing LN management that deviates from the published *CW* guidelines. Among the small proportion of MBC patients who received BCT, a consistent proportion continue to receive *CW*-discordant treatment with either ALND or no LN intervention. Furthermore, most elderly males with early-stage, node-negative, HR+, HER2− breast cancer continue to receive LN intervention above what is recommended for certain patient populations. These findings reinforce the need for additional high-level data to establish the optimal treatment strategy for males diagnosed with breast cancer to optimize patient outcomes and survival, as current treatment trends have not proven to be informed by the widely supported *CW* recommendations.
